# Assessment of the incidence and risk factors of early poststroke seizures in Lebanese patients

**DOI:** 10.1002/brb3.2204

**Published:** 2021-10-19

**Authors:** Sara Mansour, Mahmoud Youness, Sarah Cherri, Pascale Salameh, Souheil Hallit, Diana Malaeb, Hassan Hosseini

**Affiliations:** ^1^ School of Pharmacy Lebanese International University Beirut Lebanon; ^2^ Department of Neurology Al Rassoul Al Aazam Hospital Beirut Lebanon; ^3^ INSPECT‐LB: Institut National de Santé Publique Epidémiologie Clinique et Toxicologie Beirut Lebanon; ^4^ Faculty of Pharmacy Lebanese University Hadath Lebanon; ^5^ University of Nicosia Medical School Nicosia Cyprus; ^6^ Faculty of Medicine & Medical Sciences Holy Spirit University of Kaslik (USEK) Jounieh Lebanon; ^7^ Research Department Psychiatric Hospital of the Cross Jal Eddib Lebanon; ^8^ Life Sciences and Health Department Paris‐Est Créteil University Paris France; ^9^ Stroke Unit Service de Neurologie, CHU Henri Mondo ‐ 94010 Créteil Cedex France; ^10^ UPE‐C Université Paris‐Est Créteil Faculté de Santé Paris France; ^11^ INSERM U955‐E01 IMRB Créteil France

**Keywords:** ischemic stroke, poststroke seizure, early seizure, functional outcome

## Abstract

**Background:**

Early seizures have been recognized as serious complications of ischemic strokes where the data are limited among Lebanese patients. This study aimed to assess the incidence and risk factors of early seizures postischemic stroke and to determine the effect of early seizures on functional outcome among Lebanese stroke patients.

**Methods:**

This was a retrospective observational study conducted between January 2017 and March 2020 on patients with acute ischemic strokes at two tertiary hospitals in Lebanon. Data were collected from patients’ medical records at each site through a well‐designed data collection sheet. Early seizures were defined as seizures occurring within 7 days after acute stroke. Functional outcome was assessed at discharge, according to modified Rankin scale (mRS).

**Results:**

Of 140 enrolled patients, early seizure developed in 12 patients (8.6%) with mean age of 68.42 ± 9.89 years and 8 (67%) were females. Independent risk factors for early seizure development were female gender and cortical involvement. Moreover, early seizure development was not associated with higher disability and mortality at hospital discharge.

**Conclusion:**

The findings of the study highlight that early seizures occurred more commonly in patients with cortical involvement and female gender. In addition, early seizures did not impair functional outcome in our study, however; further studies are needed to predict patients at risk of early seizure so that appropriate prevention and treatment strategies can be implemented promptly.

## Graphical abstract

This is a retrospective study conducted at two Lebanese hospitals and aimed to determine the incidence, the predictive factors, and the effect of early seizures on the functional outcome of Lebanese ischemic stroke patients. Of 140 enrolled patients, ES developed in 12 patients (8.6%) with female gender and cortical involvement being the most significant predictive factors for ES. Moreover, ES development was not associated with higher disability and mortality.

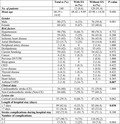

## 1 INTRODUCTION

Stroke is a cerebrovascular disorder characterized by sudden disruption of blood supply into the brain leading to injury and subsequent neurological deficit. It is estimated that the majority of stroke cases (80%) is due to the occlusion of a blood vessel by a clot resulting in an ischemic stroke (Feigin et al., 2015).

Complications frequently develop after ischemic stroke and play an important role in determining the final clinical outcome and prognosis in stroke patients. Many of the complications can be prevented through early recognition and optimal treatment which are effective strategic options in improving disease prognosis (Pezzini et al., 2013).

Seizures are one of the potentially serious neurological complications that may develop postischemic strokes (Pezzini et al., 2013). Moreover, stroke is the most common cause of symptomatic seizures and a predictor of secondary epilepsy in adults (Feyissa et al., 2019). Poststroke seizures can occur shortly after the onset of ischemia or can be delayed which explains their classification into either early or late seizures depending on the time of symptom development poststroke. According to the scientific evidence by the International League Against Epilepsy, early seizures are defined if the symptoms occur within the first 7 days after acute stroke onset and late seizures develop beyond this time window (Berg et al., 2010). Early seizures are further classified as general or partial (focal) based on the electroencephalogram findings and the clinical history (Bryndziar et al., 2016).

Studies reporting on the incidence of early seizures after ischemic strokes vary substantially which is mainly attributed to many factors including the difference in the time‐based definitions of early seizures, methodological differences in the study designs, stroke subtypes, patients’ selection criteria, and duration of follow‐up (Stefanidou et al., 2017; Wang et al., 2017; Zou et al., 2015).

The presumed pathophysiology explaining the emergence of early seizures by ischemic strokes is linked to the transient cellular biochemical dysfunctions in the brain tissue that occur following acute ischemia (Feher et al., 2020) A lower threshold for depolarization from local excitotoxicity secondary to increased glutamate along with an increased intracellular calcium and sodium inflow results in lowering the seizure threshold for depolarization and leading to electrically irritable tissue with consequent hyperexcitability and possible neuronal injury (Camilo & Goldstein, 2004).

There are many factors that are documented to increase the risk of developing early seizures postischemic stroke; cortical involvement, stroke subtype (cardioembolic infarction), hemorrhagic transformation, younger age (<65 years), and stroke severity are the most commonly reported predictive characteristics (Camilo & Goldstein, 2004; Ferlazzo et al., 2016; Lamy et al., 2003; Wang et al., 2017). Other independent predictors include alcohol intake, hyperglycemia in nondiabetics, hyponatremia at stroke onset, and large lesion size increase propensity for seizure development (Bryndziar et al., 2016; Camilo & Goldstein, 2004; Wang et al., 2013). In addition, the influence of early seizures on functional and mortality outcomes in ischemic stroke patients lead to controversial results where some studies showed that early seizures are associated with high disability and mortality rates and long‐term hospitalization, whereas others showed no association between early seizures and adverse outcomes (Burneo et al., 2010; Doria & Forgacs, 2019; Huang et al., 2014).

In Lebanon, the prevalence of stroke is high especially among elderly population and considered as the second leading cause of death after cardiac diseases among Lebanese population (Lahoud et al., 2016). Therefore, understanding the risk factors of poststroke seizures especially the early seizure type might be helpful in predicting the occurrence of seizure and thus have an impact on improving quality of life, implementing the prevention strategies, and applying timely management of early seizure.

Many studies were conducted on stroke among Lebanese patients that assessed the clinical outcomes, epidemiology, risk factors, and mortality rates (Lahoud et al., 2018; Abdo et al., 2019). However, there is lack of studies that focused on poststroke seizures and specifically early seizure. Thus, the aim of our study is to determine the incidence, the predictive factors, and the effect of early seizure on the functional outcome of Lebanese ischemic stroke patients.

### 1.1 Ethics approval

The study was approved by the Institutional Review Board of the two hospitals and by the ethics committee at the Lebanese International University. Informed consent was not obtained, as this was a retrospective study and did not pose any risk to the patients.

## 2 MATERIAL AND METHODS

### 2.1 Study design and population

This was a retrospective observational study conducted in two Lebanese hospitals. Patient's information was obtained from the medical records of each site. Two hospitals were enrolled to obtain the required cases where the number was divided almost equally between the hospitals to have a uniform patient distribution. Each involved study center generated a list of all ischemic stroke patients who were admitted to the hospital. Data were completely anonymous; names and other personal information were stripped to respect patient privacy.

### 2.2 Inclusion and exclusion criteria

All adult patients diagnosed with ischemic strokes during the period from January 2017 till March 2020 and who were subsequently admitted to the internal medicine department or intensive care unit (ICU), or cardiac care unit (CCU) were included in the study. Patients with previous history of seizures, epilepsy, transient ischemic attack (TIA), and traumatic brain injury were excluded.

### 2.3 Data collection

Medical records review was performed on site by the principal investigator who had training about the data collection, without interference or bias, using a structured data collection sheet which was divided into five sections. The first included socio‐demographic characteristics of the patients, family history, social history, and socioeconomic status. The second section included past medical history and the previous medications intake. The third section covered detailed information about stroke (first‐time or recurrent), type, lesion size, location, and complications if present during hospitalization. The fourth section evaluated stroke functional and mortality outcome at discharge through the modified Rankin scale (mRS) which is subdivided into two categories: cutoff value of 3 and above defined as a poor prognosis and ˂3 as good prognosis (Banks & Marotta, 2007; Powers et al., 2015). Also, detailed information pertinent to PSS was collected from patients` medical records through the following questions: “is the seizure new or recurrent, timing of seizure poststroke, seizure subtype, medications given to treat seizure, and anti‐epileptic drug administered at discharge to prevent recurrence.” The data collection sheet was inspired by several recent studies focusing on the pathogenesis, prevalence, risk factors, detection, management, and clinical outcome of early seizure in different stroke types (Feher et al., 2020; Huang et al., 2014; Leys et al., 2016; Zhang et al., 2014).

### 2.4 Outcomes

Primary outcome: To assess the incidence of early seizure after ischemic stroke and determine the risk factors associated with its development.

Secondary outcomes: To determine the impact of poststroke early seizure on the functional outcome at hospital discharge.

### 2.5 Statistical analysis

Statistical analysis was done using IBM Statistical Package for the Social Science software (SPSS version 25.0). Descriptive frequency tables were obtained to assess demographic data of the study population. Predictors of early seizure were first assessed by univariate analysis. Chi‐square test was used to compare patients with and without early seizure within each categorical variable. Fisher's exact test was used when the expected cell count was less than five. Multivariate logistic regression analysis was then performed with backward selection model to identify the independent predictors of early seizure. Variables with significance level of 0.2 were selected from the univariate analysis and considered for inclusion in the multivariate model. Similarly, univariate analysis was used to assess the relation between variables of interest and functional outcome. Within each of the categorical variables, including early seizure, the group of patients with Rankin <3 was compared with that of patients with Rankin ≥3 using chi‐square and Fisher's exact testing when appropriate. Multivariate analysis was also performed to identify the independent predictors of functional outcome after stroke. Missing data were not replaced since it formed less than 10% of the total data. The significance level is p‐value <0.05.

## 3 RESULTS

### 3.1 Sociodemographic characteristics

A total of 140 patients with a first‐ever acute ischemic stroke with a mean age of 66.19 ± 14.02 years and 57% males were included in the analysis. From the total enrolled patients, 12 had developed early seizure (8.6%). The mean age of the patients who had early seizure was 68.42 ± 9.89 years and 8 were females (67%). The general clinical features and risk factors of the patients with and without early seizure are summarized in Table 1.

**TABLE 1:** Distribution of ES predictors in the subgroups of stroke patients with and without ES

	Total *n* (%)	With ES *n* (%)	Without ES *n* (%)	*p*‐value
No. of patients	140	12 (8.6)	128 (91.4)	
Mean age	66.19 ± 14.02	68.42 ± 9.89	65.98 ± 14.36	.686
Gender				
Male	80 (57)	4 (33)	76 (59.4)	.081
Female	60 (43)	8 (67)	52 (40.6)	
Risk factors				
Hypertension	98 (70)	8 (66.7)	90 (70.3)	.752
Diabetes	59 (42)	3 (25)	56 (43.8)	.208
Ischemic heart disease	63 (45)	7 (58.3)	56 (43.8)	.332
Atrial fibrillation	22 (15.7)	3 (25)	19 (14.8)	.402
Peripheral artery disease	2 (1.4)	0	2 (1.6)	1.000
Dyslipidemia	59 (42)	4 (33.3)	55 (43)	.518
Current Smoking	59 (42)	5 (41.7)	54 (42.2)	.972
Cancer	4 (2.8)	1 (8.3)	3 (2.3)	.304
Previous DVT/PE	1 (0.7)	0	1 (0.8)	1.000
Sleep apnea	2 (1.4)	0	2 (1.6)	1.000
CKD	6 (4.3)	1 (8.3)	5 (3.9)	.422
Liver disease	1 (0.7)	0	1 (0.8)	1.000
Thyroid disease	6 (4.3)	1 (8.3)	5 (3.9)	.422
Anemia	2 (1.4)	0	2 (1.6)	1.000
Heart failure	5 (3.6)	1 (8.3)	4 (3.1)	.365
Asthma/COPD	2 (1.4)	2 (16.7)	0	.**007**
Clinical data				
Cardioembolic stroke (CE)	56 (40)	5 (41.7)	51 (39.8)	1.000
Non‐Cardioembolic stroke (Non‐CE)	84 (60)	7 (58.3)	77 (60.2)	
Lesion site				
Cortical involvement	55 (39.3)	8 (66.7)	47 (36.7)	.062
Length of hospital stay (days)				
≤ 7 days	89 (63.6)	4 (33.3)	85 (66.4)	.**030**
>7 days	51 (36.4)	8 (66.7)	43 (33.6)	
Stroke complications during hospital stay	102 (72.9)	11 (91.7)	91 (71.1)	.180
Number of complications				
0–2	127 (90.7)	9 (75)	118 (92.2)	
≥ 3	13 (9.3)	3 (25)	10 (7.8)	.084

Bold values indicate significance (*p* < .05)

In the univariate analysis, no differences were identified between those with and without early seizure in terms of premorbid stroke risk factors except for asthma/chronic obstructive pulmonary disease with a p‐value of 0.007. As for the duration of hospitalization, the patients with early seizure had higher length of hospital stay compared to those without early seizure (66.7% versus 33.6%; *p* =.030). On the other hand, patients with early seizure did not seem to significantly experience more stroke‐related complications during acute hospital settings than those without early seizure (91.7% versus 71.1% with a p‐value of 0.180). Our results also showed that patients with multiple complications defined by more than 3 were more prevalent in early seizure than without early seizure but the difference was not statistically significant (25% versus 7.8% with a p‐value of 0.084).

### 3.2 Assessment of the mortality and disability outcome (mRS) at hospital discharge

The analysis showed that females had significantly higher disability (mRS≥3) compared to males (59.6% versus 40.4%; with a p‐value of 0.004). As for the risk factors, patients with atrial fibrillation had significantly higher disability (mRS≥3) after ischemic stroke compared to lower disability (mRS<3) 25.5% versus 10.8% with a p‐value of 0.023. In addition, the results show that a significantly higher disability (mRS≥3) was encountered in patients with longer term of hospital stay with a p‐value <0.001 (Table 2). However, our results also showed that early seizure did not influence disability/mortality after hospital discharge (5.4% versus 14.9%, *p* =.105).

**TABLE 2:** Patient characteristics and the mortality and disability outcome (mRS) at hospital discharge

	Total *n* (%)	Rankin <3 *n* (%)	Rankin ≥3 *n* (%)	*p*‐value
No. of patients	140	93 (66.4)	47 (33.6)	
Mean age	66.19 ± 14.020	65.57 ± 13.562	67.40 ± 14.960	.357
Gender				
Male	80 (57)	61 (65.6)	19 (40.4)	.**004**
Female	60 (43)	32 (34.4)	28 (59.6)	
Risk factors				
Hypertension	98 (70)	62 (66.7)	36 (76.6)	.226
Diabetes	59 (42)	39 (41.9)	20 (42.5)	.944
Ischemic heart disease	63 (45)	40 (43)	23 (48.9)	.506
Atrial fibrillation	22 (15.7)	10 (10.8)	12 (25.5)	.**023**
Peripheral artery disease	2 (1.4)	1 (1.1)	1 (2.1)	1.000
Dyslipidemia	59 (42)	41 (44.1)	18 (38.3)	.512
Current Smoking	59 (42)	42 (45.2)	17 (36.2)	.309
Cancer	4 (2.8)	1 (1.1)	3 (6.4)	.110
Previous DVT/PE	1 (0.7)	0	1 (2.1)	.336
Sleep apnea	2 (1.4)	1 (1.1)	1 (2.1)	1.000
CKD	6 (4.3)	5 (5.4)	1 (2.1)	.664
Liver disease	1 (0.7)	0	1 (2.1)	.336
Thyroid disease	6 (4.3)	5 (5.4)	1 (2.1)	.664
Anemia	2 (1.4)	2 (2.1)	0	.551
Heart failure	5 (3.6)	3 (3.2)	2 (4.3)	1.000
Asthma/COPD	2 (1.4)	1 (1.1)	1 (2.1)	1.000
Clinical data				
With Early seizure (ES)	12 (8.6)	5 (5.4)	7 (14.9)	.105
Lesion site				
Cortical involvement	55 (39.3)	38 (40.9)	17 (36.2)	.592
Length of hospital stay (days)				
≤ 7 days	89 (63.6)	70 (75.3)	19 (40.4)	**<0.001**
> 7 days	51 (36.4)	23 (24.7)	28 (59.6)	

Bold values indicate significance (*p* < .05)

### 3.3 Multivariable analysis

When considering early seizure as a dependent variable, the multivariable analysis showed that the odds of early seizure occurrence were significantly higher in females than in males (OR 5.37). Also, the odds of early seizure increased in patients with cortical involvement (Table 3).

**TABLE 3:** Multivariate analysis: Logistic regression taking early seizure as the dependent variable

	Odds Ratio (OR)	CI (95%)	*p*‐value
Gender (Females to Males*)	5.37	1.067–27.047	.**041**
Cortical involvement (Yes versus No*)	6.388	1.274–32.037	.**024**

Note

Variables entered: gender, length of hospital stay, asthma/COPD, stroke complications during hospitalization, cortical involvement.

Bold values indicate significance (*p* < .05)

When considering modified Rankin scale as the dependent factor, the multivariable analysis showed that females and extended length of hospitalization (>7 days) had significantly higher disability (Table 4).

**TABLE 4:** Multivariate analysis: Logistic regression taking modified Rankin scales categories (mRS≥3 and mRS<3) as the dependent variable

	Odds ratio (OR)	CI (95%)	*p*‐value
Gender (Females to Males*)	2.901	1.324–6.358	.**008**
Length of hospital stay (>7 days to ≤7days*)	4.895	2.220–10.793	**<.001**
Cancer (Yes versus No*)	8.692	0.797–94.838	.076

Note

Variables entered: Early seizure, gender, length of hospital stay, cancer, atrial fibrillation.

Bold values indicate significance (*p* < .05)

## 4 DISCUSSION

This is the original study that assessed the predisposing factors to early seizure development after ischemic stroke along with the relationship between early seizure and functional outcome among Lebanese population. The main findings of this study were that early seizure occurred more commonly in patients with cortical involvement and female gender. Additionally, early seizure was not related to higher disability and mortality in stroke patients.

### 4.1 Incidence and risk factors of early‐onset poststroke seizures

Our results show that the incidence of early seizures occurring after ischemic strokes was 8.6% which is consistent with other previous studies. A study conducted by Bladin et al. reported a similar frequency of early seizure following ischemic strokes (Bladin et al., 2000). Some studies reported lower prevalence as those documented by Lamy et al. (2003), Feher et al. (2020), Stefanidou et al. (2017), and Bryndziar et al. (2016) that showed an overall frequency of 2.4%, 3.8%, 5.3%, and 7.1%, respectively. On the other hand, Misirli et al. (2006) reported a considerably higher prevalence of early seizure (10.6%) than those highlighted in our study. The wide variation in the reported early seizure frequencies in the existing literature can be attributed to heterogeneity in study designs, different early seizure definitions, and stroke subtypes included in present studies.

After multivariable analysis, females were shown to be at a higher risk of early seizure development. While most studies reported no significant difference in early seizure occurrence between both genders (Bladin et al., 2000; Burneo et al., 2010), yet there are some studies that yielded controversial results that showed the predominance of one gender (Cheung & Au‐Yeung, 2003; Kotila & Waltimo, 1992). Also, cortical involvement was shown to be a significant independent predictor of early seizures. Early seizures were significantly more frequent in stroke patients with cortical involvement than in those without cortical involvement confirming the results of most prior studies (Bladin et al., 2000; Chen et al., 2017; Lamy et al., 2003; Wang et al., 2013).

In our study, the incidence of early seizure was evenly distributed among the two subtypes of ischemic stroke with no significant difference in early seizure occurrence between the cardioembolic (CE) and noncardioembolic (large artery atherosclerosis LAA) groups. Although several studies have suggested that ischemic strokes of a cardioembolic etiology carry a higher risk of early seizures compared to atherosclerosis or other stroke subtypes (Giroud et al., 1994), however, other studies have not shown such an increased risk (Bladin et al., 2000). This is consistent with our results here where early seizure occurrence did not differ based on ischemic stroke etiologic subtype.

### 4.2 Functional outcome (using the modified Rankin scale) among patients with early‐onset poststroke seizures

Our multivariable analysis revealed that female gender and longer duration of hospitalization were significant predictors of disability following stroke in agreement with several previous studies. (Di Carlo et al., 2003; Kim et al., 2010) On the contrary, early seizures were not shown to be significant predictors of disability and mortality following stroke. To date, the influence of early seizures on functional outcome remains controversial. Some studies have reported higher disability and mortality in patients with early seizure. (Bryndziar et al., 2016; Huang et al., 2014; Jung et al., 2012; Xu et al., 2017) Burneo et al. in a recent Canadian multicenter cohort study showed patients with seizures had a greater disability at discharge compared to patients without seizures (4.41 versus 3.15, *p* =.0001). (Burneo et al., 2010) On the other hand, a number of population‐based studies did not show any association between seizures and worse functional outcome at discharge. (Alberti et al., 2008; Merlino et al., 2019) Our results in this study agree with the Dijon Stroke Registry, where early seizure did not influence short‐term disability after stroke. (Hamidou et al., 2013) Because of the relatively small number of seizure patients included in our study, additional larger studies are needed to reach a clear conclusion regarding the association between early seizure and functional outcome.

### 4.3 Limitations

The main limitations are its retrospective design and the relatively small sample size. An under or overestimation in the rate of early seizure is possible as our collected data relied on the accessible patients’ medical files. Also, our patients’ files were missing data for certain variables such as lesion size and stroke severity which limited our findings regarding the clinical characteristics of early seizures and its association with stroke severity. The evaluation of long‐term effects of early seizure on functional outcome could not be assessed in this study because patient follow‐up was not feasible with the retrospective design. In the present study, the small sample size and small number of seizure cases affected the significance of several risk factors in our multivariate analysis. Therefore, larger prospective studies are required to further evaluate the effect of the studied variables on early seizure development and the association between early seizures and functional outcome after acute stroke.

### 4.4 Impact of findings on practice statements

- This study can provide healthcare professionals with a general overview of the incidence of early seizures in our country and a better understanding of this stroke‐related complication in this population.
- Considering the risk factors of early seizures after ischemic stroke can help healthcare professionals better predict its occurrence in clinical settings which can play an important role in improving the prevention and management strategies and thereby control it.
- Given that early seizures do not seem to impact short‐term disability after acute strokes in this study, further research is needed to evaluate the effect of early seizures on long‐term functional outcome in stroke patients.

## 5 CONCLUSION

In conclusion, early seizures are common and very important complication of ischemic strokes. Cortical involvement and female gender were the most significant independent predictive factors for early seizures. Finally, early seizures did not seem to be associated with worse functional outcome at hospital discharge after acute strokes.

## CONFLICT OF INTEREST

The author(s) declare no potential conflicts of interest with respect to the research, authorship, and/or publication of this article.

## AUTHORS’ CONTRIBUTION

SM designed the questionnaire and performed data collection. Also, SM drafted and corrected the manuscript according to other authors’ suggestions. DM participated in designing the questionnaire and supervised the data collection. Also, DM supervised and corrected the study analysis plan, the statistical analysis, and the manuscript writing. SC contributed in analysis planning and in the statistical analysis. MY, PS, HH, and SH contributed in supervising the article and in reviewing the final form of the manuscript. DM and HH are last co‐authors.

## ETHICAL APPROVAL

We confirm that we have read the Journal's position on issues involved in ethical publication and affirm that this article is consistent with those guidelines.

### PEER REVIEW

The peer review history for this article is available at https://publons.com/publon/10.1002/brb3.2204.

## Data Availability

The data that support the findings of this study are available from the corresponding author upon reasonable request.
